# Perceptions and Health-Seeking Behaviour for Mental Illness Among Syrian Refugees and Lebanese Community Members in Wadi Khaled, North Lebanon: A Qualitative Study

**DOI:** 10.1007/s10597-020-00551-5

**Published:** 2020-01-21

**Authors:** Dana Al Laham, Engy Ali, Krystel Mousally, Nayla Nahas, Abbas Alameddine, Emilie Venables

**Affiliations:** 1 Médecins Sans Frontières, Operational Centre Brussels, Lebanon-Beirut-Metn-Awkar, Lebanon Mission, Beirut, Lebanon; 2 Médecins Sans Frontières, Operational Centre Brussels, Luxembourg Operational Research Unit (LuxOR), Luxembourg, Luxembourg; 3 Médecins Sans Frontières, Operational Centre Brussels, Lebanon Branch Office, Beirut, Lebanon; 4 Department of Psychology, Balamand University, Tripoli, Lebanon

**Keywords:** Mental health, Mental illness, *Jinn*, Health-seeking behavior, Stigma, Religious beliefs, Religious healers, Lebanon, Refugees

## Abstract

This is a qualitative exploration of the perceptions of mental health (MH) and their influence on health-seeking behaviour among Syrian refugees and the Lebanese population in Wadi Khaled, a rural area of Lebanon bordering Syria. Eight focus group discussions and eight key informant interviews were conducted with male and female Syrian refugees and Lebanese community members from March to April 2018. MH illness was associated with stigma, shame and fear among both populations. Beliefs surrounding mental illness were strongly linked to religious beliefs, including *Jinn*. Religious healers were considered the first line of help for people with mental illnesses, and were perceived as culturally acceptable and less stigmatizing than MH professionals. It is essential for MH professionals to build trust with the communities in which they work. Collaboration with religious healers is key to identifying MH symptoms and creating referral pathways to MH professionals in this context.

## Background

Since 2011, the conflict in Syria has had a huge impact on its population, many of whom are now displaced. The conflict has led to an enormous influx of Syrian refugees to Lebanon, the smallest country to border Syria, which globally and regionally hosts the highest number of Syrian refugees per capita (Boustani et al. 2016). According to UNHCR, approximately one million registered Syrian refugees and a further 500,000 who were not registered were distributed across Lebanon in 2018 (IOM 2019). Refugees displaced in the areas bordering Syria are considered extremely vulnerable, due to an overall lack of infrastructure and poor resources. Wadi Khaled, a rural district in the North of Lebanon, hosts about 36,000 displaced Syrians, and is one of most under-served and marginalized areas of Lebanon (Karlidage and Pineau 2017). The population is composed of different clans (  or *Ashaaer*) who share similar socio-cultural characteristics and religious beliefs. Moreover, over many decades, there have been close links, including marriage and commercial ties, between the Lebanese population in Wadi Khaled and the bordering Syrian population (Reuters 1989).

The Syrian crisis has not only affected the physical health of refugees, but has also had a drastic effect on their mental health. After experiencing war, people can develop different psychological symptoms including post-traumatic stress disorder (PTSD), anxiety and depression (Caring for Kids New to Canada and Canadian Paediatric Society 2018). After several years of displacement, some Syrian refugees have developed further psychological symptoms related to the challenging living conditions including unemployment, housing problems, education, security and discrimination in their host country (UNHCR 2013). In 2013, there was a huge need for mental health services for Syrian refugees displaced in Lebanon: according to the UNHCR, an estimated 250,000 Syrians residing in Lebanon are in need of mental health care (UNHCR 2013).

Several studies have assessed the perceptions of mental health and factors influencing access to mental health services among Syrian refugees. A study conducted in a refugee camp in Jordan showed that stigma was the main barrier for seeking mental health services and that economic instability was the main stressor affecting the mental well-being of refugees (IMC 2017). Another study conducted among Arabic speaking immigrants in Australia showed the significant influence of socio-cultural factors on health seeking behaviour for mental illness, where stigma and shame related to disclosing personal matters hindered immigrants in seeking mental health services (Youssef and Deane 2006).

However, in the unique context of Wadi Khaled, to our current knowledge, there is no published literature on the perception of mental health illness and related health seeking behaviour amongst either Syrian refugees or the Lebanese community. The population has specific socio-cultural characteristics and religious beliefs which may influence how they perceive and thus seek assistance for mental illness, including the belief in supernatural forces including *Jinn* (possession by an evil spirit), a form of black magic known as “*Sehr*”, and the evil eye or “*Hasad*”, defined as a powerful jealous look or comment upon the good fortune of another (Hanley 2005).

Healing practices to resolve psycho-social and mental health problems may also influence people’s access to mental health services (Dardas and Simmons 2015). Healing practices include prayers and reading “*Ruqya*” (specific verses from the Holy Quran), which are believed to treat spiritual aspects of illness by removing the effects of evil eye, magic, or *Jinn* possession. Beliefs in *Jinn* and its influence on health seeking behaviour have been described by scholars in several studies conducted in countries outside Lebanon (Islam and Campbell 2014; Nussrat and Simon 2018; Tzeferakos and Douzenis 2017; Weatherhead and Daiches 2010; Youssef and Deane 2006). For instance, a study that was conducted in a Syrian refugee camp in Jordan showed that mental health symptoms are considered as *Jinn* possession and can hinder the patients from accessing mental health care (IMC 2017). Similar results were shown in another study conducted in Australia, whereby Arabic speaking migrants considered evil spirits such as *Jinn or Shaytan* as reason for mental disorders and such beliefs influence patients’ health seeking behaviour (Youssef and Deane 2006).

Another study conducted in a transcultural psychiatric outpatient facility in the Netherlands showed that Muslim patients related mental illness to *Jinn* possession which affects accessing care and diagnosis by western health professionals (Lim et al. 2018).

*Médecins Sans Frontières* (MSF) has been providing mental health services to Syrian refugees and the Lebanese host population in Akkar, Wadi Khaled since 2016. In order to better understand the population we serve and provide better care, we conducted this study to understand perceptions of mental health disorders and subsequent health seeking behaviour among Syrian refugees and the Lebanese population in Wadi Khaled. This study explores factors, including socio-cultural beliefs and other factors that influence health-seeking behaviour for mental illness amongst both populations. The results of the study will help MSF and other actors to plan and provide better mental health services for such communities.

## Methodology

### Study Design

This was a qualitative research study using in-depth interviews and focus group discussions (FGDs), which was conducted in Wadi Khaled, Lebanon between March and April 2018.

### Study Setting: Lebanon

Lebanon has a total population of 4.4 million inhabitants. In addition to 1.5 million Syrian refugees, Lebanon hosts 42,000 Palestinian refugees from Syria, 6000 Iraqi refugees and nearly 450,000 refugees from Palestine (Karam et al. 2016). Mental health services in Lebanon are generally dominated by the private sector and are focused on psychiatric care. In 2014, the Ministry of Health launched a national reform of the mental health system in collaboration with the WHO, other partners and Non-Governmental Organisations (NGOs). This reform aims to integrate mental health services at primary health care level, which includes training of doctors, nurses and social workers to be able to assess, manage and refer patients to mental health specialists (Ministry of Public Health 2015). There have been challenges in implementing the program due to a lack of trained human resources, particularly in rural areas. In 2018, centres were identified for launching the new strategy in six governorates across Lebanon.

### Specific Setting

#### Wadi Khaled, Akkar

Wadi Khaled consists of 22 villages and has around 39,000 inhabitants who are predominantly Muslim. Before 1920, Wadi Khaled was socio-culturally and economically oriented towards what is currently known as the Syrian Republic, and there have been close economic, kinship and social relationships between the two (Hutson and Long 2011). With the escalation of the war in Syria and the deterioration of security in the region, Syrian refugees in Wadi Khaled, particularly those without legal resident papers and those unable to renew them due to financial reasons, have become increasingly restricted and can no longer move freely within Lebanon. Syrian refugees in this area have thus become trapped and are unable to seek health care and other basic support outside of Wadi Khaled. In the same vein, the Syrian crisis had a huge impact on the majority of the Lebanese population residing in this area including financial, social, psychological burden.

#### Mental Health Services in Wadi Khaled

MSF’s mental health services are provided free of charge for Syrian refugees and the Lebanese population in five villages in Wadi Khaled. Few actors were providing mental health support in this area. The MSF project provides counselling by psychologists, in addition to psychiatric care and community based activities. The latter (known as open groups) are conducted by the mental health team in different community venues. The aim of these groups is to raise awareness about mental health illness, build knowledge and self-help skills around psycho-social and mental health illnesses and increase self-referrals to the mental health clinic. The mental health clinic also receives cases referred from other NGOs working in the region.

### Study Population

The study population included male and female adults (aged ≥ 18 years old) residing in three villages from the areas surrounding the MSF mental health clinic in Wadi Khaled. These communities are aware and familiar with MSF activities in the region. Participants included Syrian refugees who were seeking mental health care at the MSF clinic and members of the local Lebanese community who are not beneficiaries of MSF’s mental health services. There were very few Lebanese beneficiaries seeking mental health care in the clinic during data collection of the study, and therefore, they were not included in the study. Additionally, key informant interviews were conducted with Lebanese and Syrian leaders residing in the same villages. The study included eight FGDs with a total of 46 participants, with each group having up to seven participants. The categories of participants included in the FGDs are shown in Table 1.

**Table 1:** Overview of the Focus Group Discussions (FGDs) study participants

Type of data collected	Syrian women	Syrian men	Lebanese women	Lebanese men
Number of FGDs	3	1	3	1
Number of participants	13	5	21	7

Eight interviews were conducted with key informants, ensuring participants from each village. These comprised of: one Syrian community leader (a mayor), two Syrian religious leaders (Imam and a healer), two Lebanese school principals, one Lebanese teacher, one Lebanese community leader (mayor) and one Lebanese religious leader (Imam).

The aim of having two distinctive populations for the FGDs was to illustrate any differences in the mental illness perceptions among those who are accessing mental health services and those who are in the community.

### Sampling and Recruitment

Purposive sampling was used to select Syrian participants from the MSF mental health register. The MSF psychologist contacted eligible beneficiaries telephonically to explain the purpose of the study and ensure that potential participants understood the study was not related to their routine mental health consultations. Participants were also informed that they are not obliged to participate in the study and that they had the right to refuse or withdraw at anytime. Two potential participants refused to participate when they were informed that they would not be reimbursed for their involvement. The date and time of the FGDs were arranged according to the availability of the participants. There were difficulties in recruiting men for the FGDs, as they were either at work or in search of work, and mental health was a sensitive topic for them to discuss publicly.

Convenience sampling was used to recruit FGD participants. A female MSF Health Promoter who routinely conducts awareness sessions in the local community was involved in identifying Lebanese FGD participants during her routine activities in the surrounding villages.

Purposive sampling was used to select key informants from the three villages of Wadi Khaled. Two potential participants refused to take part. All key informants were male apart from one, as it is rare to find female community leaders in Wadi Khaled. Interviews with key informants took place in offices or houses in the community, depending on the preference of the participant. FGDs with beneficiaries of MSF’s mental health services were conducted in a private room in the clinic which was not used for routine mental health consultations. FGDs with Lebanese community members were conducted at people’s houses and in one case, at the office of a mayor who was not a study participant.

### Data Collection

In-depth interviews and FGDs were conducted by the female Principal Investigator (PI) who is the supervisor of the MSF mental health program in the Akkar region, but who does not provide direct care to beneficiaries. The PI is fluent in both Arabic and English and was accompanied in all of the interviews and FGDs by a female health promoter. All interviews and FGDs were conducted in Arabic, as this was the language spoken by all participants. Interviews and FGDs took an average of 38 and 49 minutes, respectively.

All FGDs were divided by gender and nationality to ensure that participants felt at ease. Groups were divided by gender as it is culturally not appropriate to group men and women who do not know each other together in such a setting. All interviews and FGDs were audio-recorded and handwritten notes were taken by the health promoter. FGDs and interviews were conducted using interview and FGD guides that were pre-tested with local MSF staff to ensure the clarity of the questions. No reimbursements were given, but FGD participants received refreshments as this is standard practice during MSF’s community activities.

### Data Analysis

The audio-recorded files were transcribed and translated verbatim by an external transcriber. This was conducted in a “one-step” process, in which the transcriber listened to the Arabic audio files and transcribed them directly into English. The transcripts did not include any personal identifiers to protect the identity of the participants. One co-investigator who is fluent in both English and Arabic verified the accuracy of the translation and transcription by listening to the audio-recordings and reading the transcripts before verifying any discrepancies with the transcriber.

English transcripts were manually organised, coded, divided into themes and analysed by the PI and other two co-investigators who were not present during the data collection. Thematic analysis was carried out, following an approach in which the PI and a second co-investigator read the transcripts several times and independently generated codes and themes, using a coding framework based on pre-identified themes and themes emerging during data collection and interpretation. Codes were attached to quotes from transcripts in a structured manner. Differences between the two investigators were resolved through discussion. The themes and codes were finally reviewed and discussed by a third co-investigator. The interpretation of the results was also discussed with the rest of the study team throughout the study and during analysis.

## Results

The main themes identified in this study were: participants’ perceptions of mental illness; perceived causes of mental illness; barriers to seeking mental health services and alternative health- seeking behavior.

### Perceptions of Mental Illness

All participants, including those who had accessed mental health services themselves, repeatedly referred to people with mental health illness as “*stupid*”, “*crazy”*, “*complicated*” and “*weird*”.

[P]eople think that those who are receiving mental health services are crazy and they used to stay away from them. Some sympathize with them and others might say they are crazy.

30 year old Syrian man

### Causes and Origin of Mental Illness

#### Western Mental Health Interpretation

Some participants considered—“*stress*”, “*anger*”, “*fear*”, “*continuous sadness*” and “*frustration*” at their situation. Others mentioned specific mental health disorders such as depression.

[Y]ou feel sad, have a headache, shortness of breath […] you lose your appetite, you can’t work and you’re always ready to fight with anyone.

30 year old Syrian man

Depression, insomnia, involuntary and illogical acts…

Lebanese Mayor

Syrian refugees in particular talked about their sense of hopelessness and a “*fear of the future”* as a mental illness in itself:

Fear of the future and where are we going to. We were displaced: where we will go and what will happen to us?

51 year old Syrian woman

[T]his anxiety among people increased after the Syrian influx to the area. You feel that you have a fear of the future, for example, I’m alive but I don’t feel reassured.

Lebanese school principal.

#### Traditional, Religious Beliefs and Socio-cultural Context

There were similarities between the religious beliefs of Lebanese and Syrian populations residing in Wadi Khaled, as exemplified by the key informant below:

We share the same cultural beliefs as Syrians, same environment, religion, geographical areas, traditions and norms, thoughts, social behavior and demography.

Lebanese school principal

Many Syrian and Lebanese interviewees and FGD participants linked mental illness to their belief in the supernatural, demonstrating the association between mental illness and the idea of possession by evil spirits such as *Jinn*. Psychological symptoms such as sadness, depression and suicidal ideation were also described by some participants as being caused by possession. Interviewees also mentioned how the “evil eye” and feelings of envy could also influence people’s mental health:

[S]ome people behavior would make you think that it is a possession by *Jinn* and this person would be taken to a Sheikh, but in reality it is a mental health problem. When someone starts yelling on his own while sleeping; people would say that this is the *Jinn*…

25 year old Lebanese woman

[S]o her son was affected badly by the evil eye […]. He changed from being obedient to wicked […]. He became anxious and depressed.

Syrian religious leader

Although both Syrian and Lebanese participants believed that *Jinn* could play a negative role in their lives, including effecting their mental wellbeing, each group claimed that others believed more in *Jinn* and its impact on their daily life than they did:

Lebanese believe in Sheikhs more. My neighbors don’t know that I am seeing a doctor and taking pills because they don’t believe in it. […] They believe in Sheikhs.

19 year old Syrian woman

Similarly, several Lebanese participants talked about the widespread appearance of stories related to *Jinn* in Wadi Khaled since the Syrian crisis, with some mentioning that Syrians brought *Jinn* to Wadi Khaled when they were displaced to Lebanon:

I hear other people saying that the Jinn had to flee Syria due to the bombings and when you ask them where did he go? They would tell you that he left with the refugees and came to Waar region [study site], this is funny!

Lebanese school principal

Some interviewees discussed the relationship between socio-cultural norms and mental health well-being, particularly in relation to women:

A woman who lives in Wadi Khaled will suffer from depression whether she likes it or not, because she has to stay at home all day long with her children; she can’t go out. We go out on monthly basis or if there is a special occasion.

25 year old Lebanese woman

Religious origin of mental illness was mainly illustrated by some interviewees as weakness and lack of faith, as well as punishment from God:

[E]ven mental health cases are due to the weakness of faith… It happens when the person starts to behave abnormally. If the person said bad things about God, he is sure touched by Jinn, because faithful men don’t say such things.

19 year old Syrian man

[W]hen a person feels that he is missing something in his religious duties, he feels like he is getting sick.

51 year old Syrian woman

#### Contextual Factors: The Syrian Crisis, Displacement and Trauma

Syrian participants as well as Lebanese participants discussed how they believed displacement had impacted upon their mental well-being. Syrian participants cited mainly experiencing bombing and witnessing conflict; losing their homes and possessions; and the break-up of their families.

Our life is very hard. We are not happy with this lifestyle…our mental state is not well. We left our country, we left our jobs, our homes, our people and it is very hard.

60 year old Syrian woman

A school principal talked about how mental health problems among children in particular were related to the witnessing of direct conflict and death:

Syrians, especially the children, witnessed some violent acts […] We have observed some children who get scared from any noise. They are also suffering from speech disabilities due to the bombing. Others saw their mother or father dead, they saw blood.

Generally, the Syrian crisis and the forced displacement had a prominent effect on unemployment, poverty and inadequate housing in the region, which were linked by participants to the mental health problems in the community. Syrian refugees in particular discussed a loss of hope, lack of independence, sense of isolation and feelings of being trapped.

We used to be stable in Syria and my husband used to work; now my husband doesn’t work and two of my children are sick. We have to move from place to another.

30 year old Syrian woman

[W]hen a man comes home and his son asks for money, and the father doesn’t have any as he doesn’t work and is sick, it makes him feel bad, he might leave the house and cry.

38 year old Lebanese male

Families are separated now; we can’t go to Beirut or the South because of the residency permit. We are restricted to one place and this is a source of pressure.

41 year old Syrian male

The majority of Lebanese participants discussed the huge burden of the Syrian crisis on their daily life. For instance, some related lack of employment and financial difficulties to the fact that Syrians are competing with Lebanese in the labor market in Wadi Khaled.

Our village has always lacked job opportunities, but with the Syrian influx, job opportunities have vanished.

48 year old Lebanese woman

Additionally, some of the Lebanese participants highlighted negative impact on the social and psychological wellbeing of the Lebanese and the increase rates of violence.

Our problems increased with the Syrian influx: crimes, stealing, rape and others. The end result is psychological stress.

25 year old Lebanese woman

### Barriers to Seeking Mental Health Services

There were several reasons for not seeking mental health services, including a lack of knowledge about mental health symptoms and available services, stigma and financial barriers.

#### Lack of Knowledge About Mental Illness and Services

Several interviewees discussed the general lack of knowledge about mental illness within their communities and how symptoms were usually linked to *Jinn.* Some also expressed the lack of awareness about existing mental health providers and services in their communities.

They said she is touched or something, they took her to Sheikh as they didn’t know about the psychiatrist and up till now the majority do the same.

36 year old Syrian woman

They don’t even know to whom they should go if they are suffering from psychological illness.

Lebanese school principal

Additionally, some of the interviewees who sought mental health care mentioned their experience with the misinterpretation of the mental health symptoms among health providers.

I visited a doctor, but when I told him I am tired and have a headache he told me all of us feel like this. He could have said that my blood tests are normal and that I should see a psychiatrist, especially as he knows that I am Syrian.

40 year old Syrian male

On the other hand, participants described an overall lack of services in Wadi Khaled, including those for mental health, which was worsened by the geographical isolation, with one teacher talking about how people are “*far from the city, difficult roads and there is lack of services*”.

#### Stigma, Shame and Fear

Feelings of embarrassment, fear and stigmatization were perceived by the interviewees as a hurdle to seek mental health services. Some interviewees explained how people seeking mental health services would be mocked and labeled in the community as it was considered “taboo” and someone would be described as “crazy”.

One Syrian participant described how he preferred to tell his parents that he is seeing a doctor instead of using the word “psychologist”:

My family only knows that I am having a therapy… They don’t ask me to specify if I am going to a psychologist, so I just say I am going to a doctor.

A Syrian woman described a similar experience:

[T]hey will call us crazy and people won’t come to us anymore. They say we might hurt them. This is why we come [to MSF clinic] without telling anyone.

#### Financial Barriers

Lack of financial support and poverty were considered significant obstacles to seeking general health-care as well as mental health services. These concerns were mainly focused on the cost of transport, consultations and treatment.

[M]oney […] I saw doctors in Wadi Khaled and they wanted to transfer me to a psychiatrist in Tripoli, but I couldn’t because of the lack of money, and they tell you that the therapy will last minimum a year and that you have to buy medications every month.

30 year old Syrian woman

### Health-Seeking Behavior and Role of Religious Healers

There were many examples of experiences of mental illness which were perceived by the Syrian and the Lebanese participants to be linked to *Jinn*, including their description of the pathway of care, the role of religious healers, the use of Ruqya which consists of reading verses from the Quran and the use of practices such as “hitting a body part” as a practice for exorcism when a person is possessed by *Jinn*.

[S]ome people get nervous and have seizures. They say she is touched by Jinn, so they take her to the Sheikh. No one thinks that she might be sick and need a doctor […] A woman died because of this.

36 year old Syrian woman

[H]e took the knife and wanted to cut his penis, I asked him “why?” He said someone told him to. I was shocked. His brother calmed me down, we didn’t sleep, his brother and I monitored him… We were afraid he could commit suicide. First we took him to a sheikh, he hit him on the stomach. It took him a whole year with the sheikh. He didn’t feel better except with medication… I borrowed money and took him to a doctor in Tripoli who gave him treatment.

55 year old Lebanese woman

[T]he person shouts and break things at home and his family sought help at a “legal” sheikh. The sheikh knows and he read the Quran for him. If there is really *Jinn*, it burns… I witnessed that […]. This has no relation with a psychiatrist, it is a *Jinn* case.

19 year old Syrian male

Participants discussed how the first step in seeking medical or mental health services is often to go to a religious leader, before seeking assistance elsewhere if there was no improvement. One Lebanese woman stated how “*if the Sheikh couldn’t help, we could take him to a psychologist*.”

Some participants described their personal experiences with religious healers who diagnosed them as being possessed, and talked about how their conditions worsened.

I talked to a friend, who sent me to a sheikh and this sheikh made me feel crazier. The sheikh tells that there is magic, but the fact it is all about psychology, when you think of what the sheikh tells, you start believing that someone had done magic on you, you get more complicated  , I got more complicated. I went to 3 or 4 sheikhs and they made me feel sicker […] Some read the Quran for me and others said that someone did magic on me, this made me more tired and sick.

30 year old Syrian woman

Sheikhs used to warn me about going to a psychiatrist and receiving any medications.

30 year old Syrian woman

[T]hey use very harmful techniques to try expelling the “devil” from the body; they harm the person physically and make him feel even worse.

School principal

Whilst many participants preferred seeking help from religious healers, there were some people who did not trust them:

[W]e can’t call these people religious figures, they are not well educated and they consider it as a business, so that they don’t allow people to go to doctors. You can call them magicians not sheikhs.

51 year old Syrian woman

[R]eal sheikhs don’t say that you are touched by *Jinn*; they only read Quraan for the person […] that’s his duty.

19 year old Syrian man

[S]ome sheikhs are not real, whereas others are very helpful and they try to do everything to help…..True religious figures are those who follow Quraan and Prophet…other than this they are only fooling people” ….

21 year old Lebanese woman

Some participants, including a Syrian Mayor, also described the importance of having a social network support of trusted family and friends which could function as an alternative form of support for mental illness.

## Discussion

This study presents an in-depth analysis of the perceptions of mental illness among Syrian refugees and Lebanese community members in the rural area of Wadi Khaled, Lebanon. To our knowledge, this is one of the first studies in the north of Lebanon highlighting people’s perceptions of mental illness, particularly the distinct socio-cultural and religious beliefs which influence people’s health-seeking behaviour. These results are currently of particular significance as the Lebanese government has begun to scale-up mental health reform across the country, in response to the huge mental health needs among Syrian refugees and the Lebanese population.

Mental illness was generally associated with shame, stigma and fear by both Syrian and Lebanese populations. It was evident during all the FGDs and interviews that mental health was a sensitive subject as it is highly stigmatised and brings shame to those affected and their families. These results are similar to other studies that explored mental health perceptions among Syrian refugees in the Middle East and Europe (Ben et al. 2018). However, a recent study by Karam et al. among Lebanese participants who sought mental health care showed that stigma was reported by less than 6% of them as a barrier to seeking help (Karam et al. 2018). This contradiction could be explained by the specific socio-cultural context in the extremely rural area of Wadi Khaled.

People with mental illness were often considered to be dangerous by others, were perceived to be untreatable and thus to be avoided in the community. The origin of fear has been linked to lack of knowledge of severe mental disorders such as schizophrenia and bipolar disorder and mild to moderate psychological symptoms such as anxiety (Youssef and Deane 2006).

Our study participants strongly linked mental illness to the presence of *Jinn*, and religious beliefs played a clear role in how mental illness was perceived and how it was treated.

Our data showed that many Lebanese community members believed that practices related to *Jinn* had increased in Wadi Khaled since the displacement of Syrians  which could be translated as “Black magic”, was also believed to be commonly used in Wadi Khaled by both populations, they related changes in life or personality to “magic spells” which can be detected and healed by consulting a religious healer. Magic spells were believed by the interviewees to be healed by religious healers. This has often led to avoiding mental health providers and implied drastic drawbacks on the people suffering from mental illnesses, particularly those with severe conditions. The correlation between *Jinn* and mental illness has been found in many studies. For instance, similar results were observed in a study among Muslim communities in the United Kingdom, in which differences between Western and Islamic understandings of mental distress were shown to influence the ways in which people sought help, with Muslim communities shown to seek help through prayer, consultations with religious leaders and family support rather than from health-care professionals (Weatherhead and Daiches 2010).

Our results show how the geographical isolation of the Wadi Khaled area, economic challenges and unemployment associated with a fear of the future and hopelessness were also believed to be causes of mental illness. The Syrian crisis and massive displacement of people were also considered to be a significant trigger for symptoms of mental illness. This link has been noted elsewhere, with a similar study in a refugee camp in Jordan also showing how Syrian refugees linked mental illness to wider social factors (IMC 2017).

There were several barriers to seeking mental health services in Wadi Khaled, including a lack of knowledge of mental health symptoms and services, a fear of stigmatisation and economic concerns. People were afraid of being discriminated against, stigmatized and marginalized if they consulted mental health services, and beneficiaries who sought mental health care tried to ensure that their consultations were not publicly known, even among their family, for fear of being labelled “crazy”. Shame and fear played a key role in hindering people with mental health illnesses from accessing care. Interestingly, similar findings have been noted elsewhere, including amongst Arabic speaking communities in Australia, in which similar beliefs were also found to hinder access to mental health care (Youssef and Deane 2006).

Seeking help from religious healers for mental illness was believed to be less stigmatising and more culturally acceptable than seeking assistance from a mental health provider such as MSF. This is may be due to the predominant beliefs which are deeply linked and rooted to religion, and the fear of stigma associated with seeking help from mental health providers.

Seeking help from family and friends was considered an acceptable alternative to seeking mental health care services from MSF. A study exploring the health seeking behaviour of refugees in Lebanon showed that Syrian refugees would rather seek help from their general practitioners in private or poly-clinics rather than from MH professionals, which was not the case in our study (UNHCR 2013). Another study showed that Syrian refugees in Jordan refugee camps would seek help for mental health issues within their own community to avoid being stigmatised as they seek professional MH care (IMC 2017).

Our study had a number of limitations. In a context with such economic hardship, it was challenging to recruit men for FGDs as they were working or seeking employment. Our study did not include Lebanese participants seeking MH care at our clinics, but during the FGDs some Lebanese participants expressed their experiences of seeking MH care outside of MSF. The PI’s role as a mental health provider and presence during data collection may have biased the participants’ responses, which is often unavoidable in qualitative research (Anderson 2010; Ochieng 2009). However, this study, to the best of our knowledge, is the first study that tackled mental illness in relation to cultural beliefs amongst Lebanese and Syrian refugees in the Wadi Khaled region. It illustrated how the mental health seeking behaviour of this population is influenced by the religious leaders of that area and cultural perceptions. It also provided us with a better insight on how to improve interventions in our mental health project in the Akkar region.

Several policy implications and recommendations can be drawn from this study. Firstly, at the community level, there is a vital need for an adapted awareness campaign about mental health symptoms as well as services in the region, focussing on households and schools. Although MSF is providing community awareness and psycho-education sessions in Wadi Khaled, there is still a need to integrate certain aspects relating psychotherapy to socio-cultural and religious beliefs. These messages need to be adapted to the specific context of Wadi Khaled in collaboration with influential religious leaders who are aware of the socio-cultural context and who are well-known within their communities. The approach should focus on decreasing stigma by illustrating the difference between interpretations of mental illness, considering the cultural/religious perceptions of mental illness versus the western interpretations, and through common language that relates traumatic experiences to mental illness. Anecdotal evidence shows that normalizing symptoms has proved to be very helpful in accessing mental health services and thus reducing stigma.

Secondly, engaging community leaders and raising their awareness about mental health illness is fundamental. An open dialogue between mental health providers and these stakeholders is recommended. Further research is still required to fill the gap of knowledge related to the benefits and risks of integrating specific religious interventions to the evidence-based psychological intervention for certain mental disorders. Thirdly, as highlighted by teachers and school principals, schools could be the gateway to accessing the community through identifying and supporting the mental health needs of children as well as parents.

Finally, as the government is in the process of launching mental health care centres across Lebanon, it could be of great importance to ensure attention is given to remote areas such as Wadi Khaled.
